# Properties Optimization of Polypropylene/Montmorillonite Nanocomposite Drawn Fibers

**DOI:** 10.3390/nano14020223

**Published:** 2024-01-19

**Authors:** Konstantinos Leontiadis, Katerina Theodoratou, Costas Tsioptsias, Ioannis Tsivintzelis

**Affiliations:** 1 Department of Chemical Engineering, Aristotle University of Thessaloniki, University Campus, 54124 Thessaloniki, Greece; 2 Department of Chemical Engineering, University of Western Macedonia, 50132 Kozani, Greece

**Keywords:** polypropylene, montmorillonite, Cloisite^®^, fiber, drawing

## Abstract

In this study, the mechanical properties and thermal stability of composite polypropylene (PP) drawn fibers with two different organically modified montmorillonites were experimentally investigated and optimized using a response surface methodology. Specifically, the Box-Behnken Design of Experiments method was used in order to investigate the effect of the filler content, the compatibilizer content, and the drawing temperature on the tensile strength and the onset decomposition temperature of the PP composite drawn fibers. The materials were characterized by tensile tests, thermogravimetry, and X-ray diffraction. Two types of composites were investigated with the only difference being the type of filler, namely, Cloisite^®^ 10A or Cloisite^®^ 15A. In both cases, statistically significant models were obtained regarding the effect of design variables on tensile strength, while poor significance was observed for the onset decomposition temperature. Nanocomposite fibers with tensile strength up to 540 MPa were obtained. Among the design variables, the drawing temperature exhibited the most notable effect on tensile strength, while the effect of both clays was not significant.

## 1. Introduction

Polypropylene (PP) is one of the most commonly used thermoplastics. Chemical inertia, easy processing, good mechanical properties, low cost, and high thermal stability are some of the polypropylene’s attractive properties. PP is mostly used in the forms of sheets or filaments. The former are generally used in packaging, while the latter are used mainly for fibers and textiles [1]. Due to the commercial importance of PP, it is essential to further improve its mechanical and thermal properties. A well-established and common practice to enhance the properties of polymers is the development of composite materials via the addition of fillers into the polymer matrix. The drawing of filaments/fibers leads to the formation of anisotropic structures, due to the alignment of macromolecules and crystallites in the direction of drawing, resulting in a considerable increase of the mechanical strength of the drawn filaments/fibers. In this direction, the combination of drawing and utilization of fillers has attracted much research attention, and the results of this combined approach to improving PP composite drawn fibers have been recently reviewed [1].

Organically modified montmorillonite (MMT) is one of the most common fillers utilized in the production of nanocomposite PP materials [1]. In cases of poorly polar polymers, such as PP, the modification of MMT may not be adequate to induce sufficient favorable intermolecular interactions with the polymer matrix. Thus, it is common to additionally use compatibilizers, e.g., maleic anhydride grafted polypropylene (PP-g-MA). Layered silicates, such as MMT, can form three different types of structures inside a polymer matrix. The first type forms when macromolecules cannot penetrate the interlayer space. This is called a microcomposite material because the filler aggregates are, in general, greater than 100 nm. The second possible structure, the so-called intercalated structure, is the result of the penetration of some polymer chains into the interlayer space. The chains do not fully destroy the layered structure of the filler, but they do increase the interlayer space. The existence of such structures is revealed in XRD diffractograms as a shift to lower angles of the characteristic peak related to d_100_ distance. The third type, called an exfoliated structure, is achieved when the polymer chains penetrate the interlayer space and fully separate the filler’s layers. The last two structures are considered actual nanocomposite structures.

As in the case of other polymers, MMT has been utilized for the development of polypropylene composites, e.g., Joshi et al. examined the effect of 0.25 and 0.5 wt.% Cloisite^®^ 15A with a compatibilizer in ratios of 1:1, 1:2, and 1:3 with respect to the filler [2]. As mentioned above, results regarding PP composite drawn fibers have been recently reviewed. However, a comparison of composite samples, even those with the same filler (e.g., MMT), is not simple, since the various samples have been subjected to different drawing ratios or other processing conditions. Nevertheless, it is clear that the drawing ratio heavily impacts the properties of the composite PP fibers, as in the case of pristine PP fibers [3,4]. In another study, the drawing capability of polypropylene fibers filled with organically modified montmorillonite or hydrotalc modified with fatty acids was investigated. PP-g-MA was used as compatibilizer. The results revealed that the composite fibers exhibited increased thermal stability, while the mechanical properties were similar to that of the pristine PP fibers. The composite fibers showed increased drawing capability [5]. Montmorillonite modified with cetyltrimethylammonium bromide (CTAB) was also used as a filler to produce PP drawn fibers. The nanocomposite fibers showed a doubled Young’s modulus and an increase in tensile strength from 532 MPa to 690 MPa [6]. Cloisite^®^ 20A was used as a filler, without a compatibilizer, resulting in enhanced properties. However, the improvement was attributed to the increased drawing ability, rather to the direct effect of the filler [7]. Another study on nanocomposite PP-Cloisite^®^ 20A fibers showed that the use of the filler resulted in a less rough fiber surface and a more uniform fiber diameter [8]. The compatibility between filler and polymer are important [9] and for the case of PP, it has been reported that modification of both MMT and polypropylene is desirable [10].

In our previous works [3,4], we used various inorganic fillers (wollastonite, attapulgite, talc) and carbon nanotubes to enhance the mechanical and thermal stability of PP drawn fibers. In such experiments, a commercial antioxidant and a compatibilizer were also used. The optimization of the properties through surface response analysis revealed that the effect of drawing is considerably higher than the effect of the fillers (as in the case of Cloisite^®^ 20A [7] mentioned above). In most cases, a maximization of tensile strength could be achieved without using any filler. Besides drawing, other effects, e.g., strong favorable interactions of the compatibilizer with the antioxidant (instead of interactions with the filler), were found to contribute to the absence of countable effects of fillers on mechanical and thermal stability. Though the particles in some cases were nano-sized particles, the above-mentioned composites were not actual nanocomposites. Thus, in this study we aimed to develop nanocomposites in order to further improve the mechanical and thermal stability of PP drawn fibers. For this reason, we examined two different modified MMTs. More specifically, polypropylene drawn fibers containing compatibilizer (PP-g-MA) and either Cloisite^®^ 10A or Cloisite^®^ 15A were produced. The experimental runs were determined by the experimental matrix produced using the Box-Behnken methodology. Mechanical and thermal stability were examined via tensile tests and thermogravimetry (TGA). Optimization of the properties was also performed.

## 2. Experimental

### 2.1. Materials

Isotactic PP (Ecolen HZ42Q) with a melt flow index equal to 18 g/10 min, a tensile strength equal to 33 MPa and a melting point of 168–171 °C was obtained from Hellenic Petroleum S.A, Thessaloniki, Greece. A masterbatch (Bondyram^®^ 1001) with PP grafted with maleic anhydride (PP-g-MA), with an MA content equal to 1%, melt flow index equal to 100g/10 min, and melting point of 160 °C, was obtained from Polyram Plastic Industries Ltd., Gilboa, Israel and used as a compatibilizer. A masterbatch with an antioxidant was also used (KRITILEN^®^ AO PP9216, Plastika Kritis S.A., Heraklion, Greece). Organically modified montmorillonite (Cloisite^®^ 10A and 15A) was purchased from Southern Clay Products Inc., Austin, TX, USA. In Table 1, important characteristics of the used materials are presented.

### 2.2. Fiber Production and Drawing

A KERN PLS 1200–3A scale (±0.003 g) was used for preparing the pellet-powder mixtures. In all cases, pellets of isotactic polypropylene were mechanically pre-mixed with pellets of the compatibilizer’s masterbatch (PP-g-MA), antioxidant’s masterbatch, and organically modified montmorillonite in powder form. In order to produce the drawn fibers, the procedure that was followed involved two extrusions and a solid-state drawing. Before the extrusions, Cloisite^®^ powder was heated in an oven at 80 °C for 8 h. Initially, the mixture of PP and additives (compatibilizer, antioxidant and Cloisite) was introduced in a twin screw (screw rotating speed equal to 25 rpm) extruder (HAAKE Rheodrive 5001, Thermo Fisher Scientific, Waltham, MA, USA) with four heating zones (190, 210, 215 and 220 °C from feed to nozzle). The produced filament was cut into pellets and was fed into a single-screw (screw rotating speed equal to 15 rpm) extruder (Noztec Xcalibur, Shoreham-by-Sea, UK) with three heating zones (215, 225 and 210 °C from feed to nozzle). A winding machine (Noztek Filament Winder 2.0, Shoreham-by-Sea, UK) was used to collect the produced fiber into a drum. Finally, solid-state drawing was performed in a homemade apparatus at various temperatures, by keeping the drawing ratio constant at 7. After drawing, the produced fibers had a diameter between 150 and 250 μm, depending on the diameter of the initial filaments. All samples contained 2 wt% of the antioxidant’s masterbatch. More details can be found in our previous studies [3,4].

### 2.3. Characterization

The thermal properties of the samples were studied via thermogravimetric analysis (TGA) using a Shimadzu TGA-50 thermogravimetric analyzer (Shimadzu, Tokyo, Japan). TGA measurements were performed with a heating rate of 20 °C/min up to 600 °C in a room-air atmosphere. The onset decomposition temperature was declared to be the temperature at which the remaining mass was equal to 97% of the initial mass.

The mechanical properties were studied via tensile tests using a Hans Schmidt & Co GmbH Universal Testing Machine ZPM (Waldkraiburg, Germany), equipped with a Pacific PA6110 load cell (head speed 100 mm/min). For each sample at least 15 tensile tests were performed, and the values presented in the next sections are the average values ± the standard deviation of all measurements.

In order to study the structure of the fibers, XRD analysis of some selected samples was carried out. Specifically, a Bruker D8 Advance (Billerica, MA, USA) diffractometer equipped with a Siemens X-ray tube (Cu, 1.54 Å) was used. Three types of samples were examined: fibers, films, and powders. Powder was used without further treatment. Fibers (drawn and not drawn) were cut in pieces in order to fill the sampler. Pristine polypropylene was formed into a film through a heat press. The thermal plates of the press were set at 170 °C. When the plates reached the aforementioned temperature, pressure of 100 bar was applied to the sample for 15 min. After 15 min, the heating was turned off and the sample was allowed to cool under pressure. When the heat plates reached room temperature, the pressure was decreased and the produced film was used for XRD analysis. For all XRD measurements, all samples (powder, film, or fibers) were examined from 2 to 30°.

## 3. Design of Experiments

Optimization of the properties of the composite drawn fibers was carried out using a surface response methodology, through a Design of Experiments (DoE) using the Box-Behnken method. Minitab^®^ 20.4 software was used. The plots presented in the next sections were reproduced using the Python libraries NumPy [11] and Matplotlib [12].

Two experimental designs were used. The factors and the factor levels were the same for both. Specifically, the filler content (0–2 wt%), the content of compatibilizer’s masterbatch (0–4 wt%), and the drawing temperature (100–140 °C) were used as factors (design variables). The only difference was the type of the filler used (Cloisite^®^ 10A for the first design and Cloisite^®^ 15A for the second). The resulting experimental matrix is shown in Table 2. The matrix was produced based on the above choices using the Box-Behnken Design of Experiments method. The response variables chosen for both designs were the tensile strength and the onset decomposition temperature (considered as the temperature in which the remaining mass is equal to 97% of the initial mass). The optimization method was based on the maximization of composite desirability.

Before proceeding with the presentation and discussion of the results, it is worth mentioning the rationale for selecting the ranges of the design variables. In microcomposites, in order to achieve a countable alteration of the polymer’s properties, it is common to use high additive contents, e.g., 5–15%. By contrast, in nanocomposites, due to the increased surface area of the filler, the enhancement of properties can be achieved using much lower additive contents, as low as 0.05%. In addition, it must be taken into account that an increased filler content makes the formation of aggregates more probable. Aggregates are undesirable in the drawing process. For these reasons, the Cloisite^®^ content was studied in the narrow range of 0–2 wt%, which is a typical range for polymer nanocomposites.

The range for the compatibilizer masterbatch (0–4 wt%) was selected based on the typical industrial practice and on a previous study for PP-wollastonite drawn fibers [3]. In that study, compatibilizer masterbatch contents up to 15 wt% were investigated. It was concluded that high compatibilizer contents have a negative effect on tensile strength, because the stretching of chains is hindered due to the low molecular weight of the compatibilizer.

Finally, for the drawing temperature, the range was selected on aspects related to PP’s thermal behavior. For the drawing of semi-crystalline polymers with a glass transition temperature (*T_g_*) higher than room temperature, the *T_g_* is very important for selecting the temperature of drawing. For polymers like PP, with a *T_g_* lower than room temperature, drawing can be performed at room temperature, subject to various limitations, including a low drawing potential, a high tendency to shrink after drawing, etc. [1]. For such polymers, the crystallization temperature (*T_c_*) is more important. Drawing can induce crystallization, which is facilitated by higher temperatures. On the other hand, if drawing occurs at temperatures lower than but close to *T_c_*, crystallization is not totally precluded, but the lower temperature may offer additional benefits, such as faster cooling after drawing and thus lower relaxation and shrinking before the structure manages to stabilize. Of course, the crystallization temperature depends on the thermal history of the material, but typical values for PP are in the range 110–120 °C. Thus, the selected range of 100–140 °C for the drawing temperature is a range of values roughly equal to *T_c_* ± 20 °C.

## 4. Results and Discussion

Results for DoE with Cloisite^®^ 10A and 15A are presented in the next two subsections.

### 4.1. DoE with Cloisite^®^ 10A as a Filler

In Table 3, the factors and responses for the DoE using Cloisite^®^ 10A as a filler are presented. The tensile strength and the onset decomposition temperature (*T_dec_*) were used as response variables. Typical stress-strain and TGA graphs are presented in Figures S1 and S2 of the Supplementary Information File, respectively.

#### 4.1.1. Main Effects of the Factors

**Figure 1:**
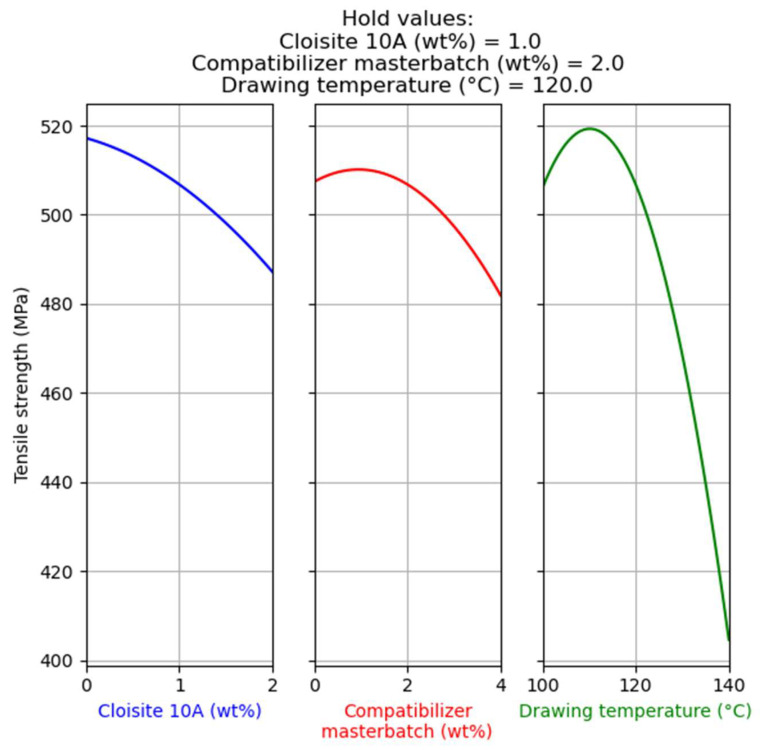
Main effect of factors on tensile strength of the composites with Cloisite^®^ 10A.

The main effects of the factors on the tensile strength are presented in Figure 1. Each graph shows the pattern which each response variable follows when a specific factor is examined, while the other two factors have fixed values (hold values). For example, for the left graph of Figure 1 the compatibilizer’s masterbatch content is equal to 2 wt%, while the drawing temperature is equal to 120 °C. In every plot, the hold value of each factor is equal to the middle value of the examined range.

As presented in Figure 1 (plot on the left), the addition of Cloisite^®^ 10A has a mild negative impact on tensile strength. A similar trend is shown for the compatibilizer content (plot in the middle of Figure 1). Both of these effects can be attributed to the hindering of the drawing process. As concluded by our previous studies [3,4], drawing is the dominant factor in terms of tensile strength improvement in composite fibers. If the filler hinders the drawing process (i.e., the alignment of macromolecules and crystallites), or if the filler results in high crystallinity prior to the drawing process (due to heterogeneous nucleation), it leads to a decrease of tensile strength. Furthermore, the compatibilizer has a lower molecular weight, which does not favor the drawing process [1]. However, as revealed by a previous study, the compatibilizer contributes to an improvement of antioxidant dispersion in the polymer matrix [3,4]. The slight increase in tensile strength for compatibilizer contents up to 1 wt%, seen in the middle plot of Figure 1, can be attributed to this effect. Raising the drawing temperature up to around 110 °C results in an increase of tensile strength. Above this temperature, a steep decrease of tensile strength is observed (plot on the right of Figure 1). This can be attributed to the dual effect of temperature. The increase of temperature facilitates the rearrangement of the macromolecules and thus the chain alignment during drawing, resulting in the increase of tensile strength observed up to approximately 110 °C. However, right after the drawing, the shrinking of fibers that is thermodynamically favored becomes more kinetically feasible at higher temperatures (during the cooling of the fibers) and thus more intense, resulting in the partial destruction of the produced aligned structure. The latter effect seems to dominate above 110 °C, resulting in a step decrease of tensile strength.

The effects of the main factor on the onset decomposition temperature are presented in Figure S3 of the Supporting Information File. Focusing on the range of the *y*-axis of Figure S3, it is apparent that none of the factors contributes significantly to the onset decomposition temperature and, as will be shown in the next section, the model that is created is not statistically significant. Therefore, this response variable will not be examined further. The rest of this paper focuses on tensile strength.

#### 4.1.2. Combined Effect of the Factors

**Figure 2:**
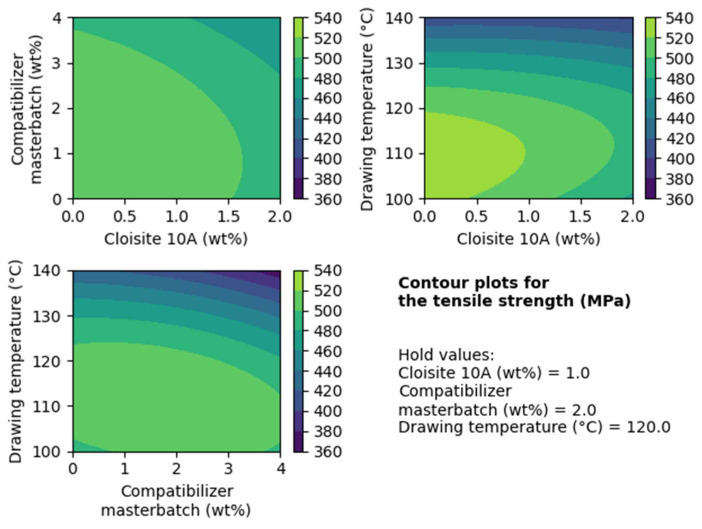
Contour plots for the tensile strength regarding the composites with Cloisite^®^ 10A.

In Figure 2, the contour plots for the tensile strength are presented. In each of these plots, the effects of two factors on the value of the tensile strength are presented, while the third factor is kept constant (hold value).

Based on these contour plots, it is obvious that filler contents of less than 1 wt% offer the highest tensile strength. The same applies to compatibilizer contents equal to or less than 2 wt% A drawing temperature of 110 °C yields the best results.

#### 4.1.3. Optimization

**Figure 3:**
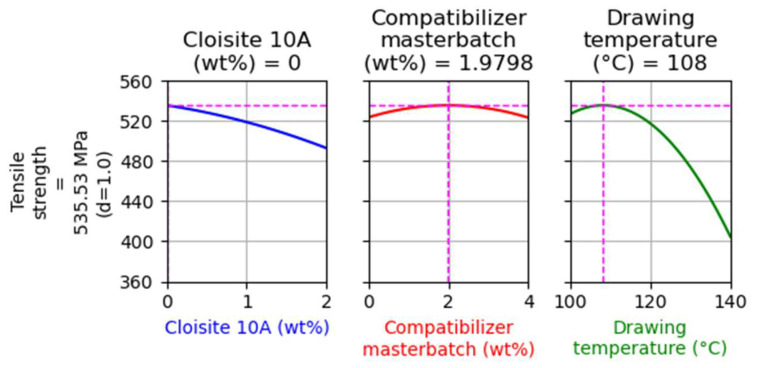
Optimization targeting the maximization of tensile strength without restrictions for the case of composites with Cloisite 10A.

**Figure 4:**
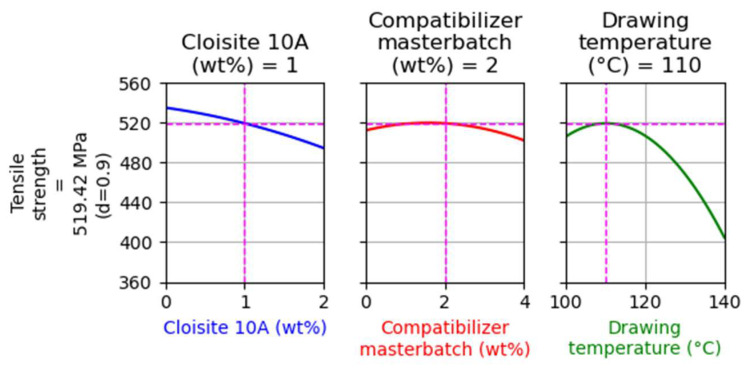
Optimization targeting the maximization of the tensile strength using as constraints the filler and the compatibilizer content to be equal to 1 and 2 wt%, respectively.

For this DoE (regarding the composites with Cloisite^®^ 10A), two optimizations were performed targeting the maximization of tensile strength. The first optimization had no restrictions, while in the second the filler and compatibilizer content were kept equal to 1 and 2 wt%, respectively. These optimizations are presented in Figure 3 and Figure 4, respectively.

As shown in Figure 3, the maximum tensile strength is achieved without any added filler, with the addition of 2 wt% of the compatibilizer masterbatch, and using a drawing temperature of 108 °C. The predicted tensile strength is approximately 535 MPa.

As presented in Figure 4, the addition of filler at 1 wt% did not cause a large decrease of tensile strength, though a small reduction of about 15 MPa compared to the results of Figure 3 was observed. This sample was selected to be further examined in order to evaluate the prediction ability of the model.

#### 4.1.4. Model Evaluation

In order to confirm the prediction ability of the model, a sample with the composition shown in Figure 4 was produced and submitted to tensile tests and TGA. The same sample was also examined using XRD before and after drawing. In Table 4, the experimental results and the model’s predictions are presented along with the absolute relative deviations.

The relative deviations between predictions and the experimental results are low, around 8% for the tensile strength and 2% for the decomposition temperature. The statistical significance of the model is evaluated through the *p*-value and R^2^, which are presented in Table 5.

Based on the R^2^ and *p*-values, the tensile strength model is statistically significant. The model for the decomposition temperature presents poor results. However, the prediction of the decomposition temperature showed a low deviation from the experimental measurement. This is a result of the narrow range of the responses (290–302 °C, see Table 3), which means that every value that falls into this range will present a low absolute deviation.

#### 4.1.5. Fiber Structure

**Figure 5:**
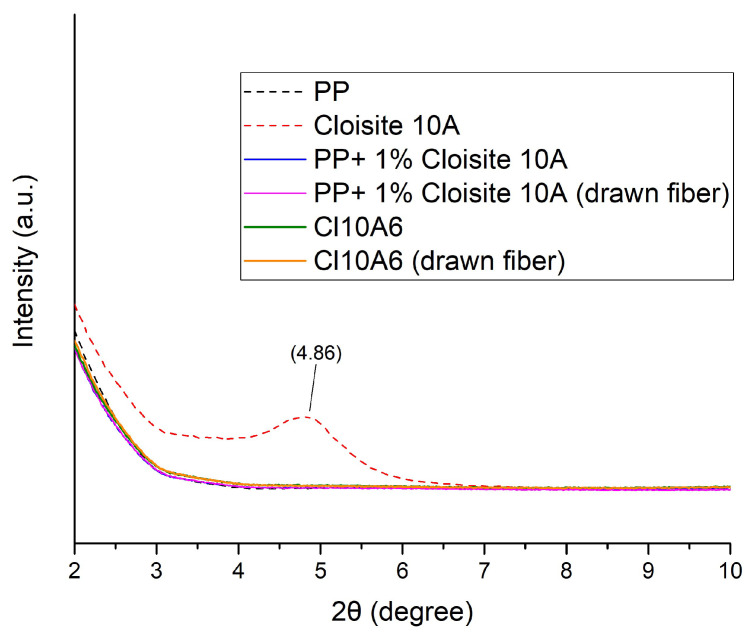
XRD diffraction patterns of PP, Cloisite^®^ 10A, two composite PP fiber samples containing 1 and 2% Cloisite^®^ 10A, drawn and not drawn, at lower angles (2–10°).

**Figure 6:**
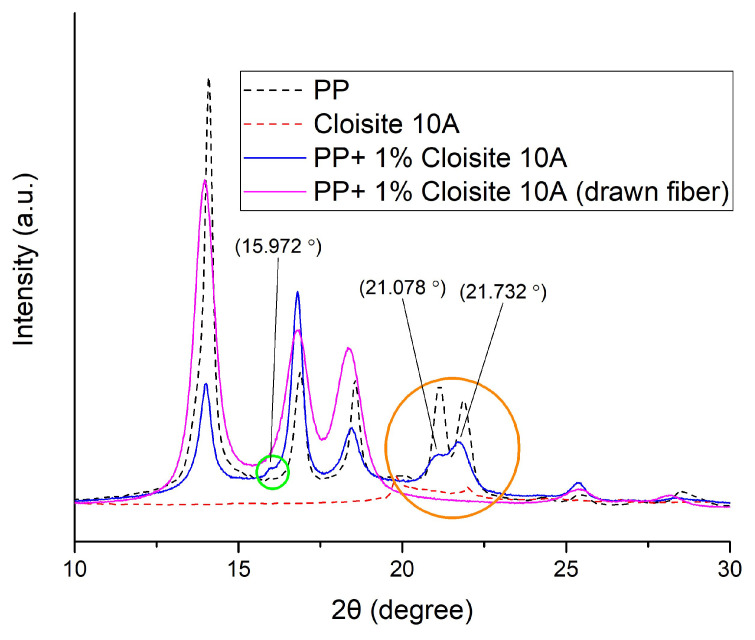
XRD diffraction analysis of PP, Cloisite^®^ 10A, and a composite PP fibers containing 1% Cloisite^®^ 10A, drawn and not drawn, at higher angles (10–30°).

In order to examine the structure of the drawn PP-Cloisite^®^ 10A fibers, XRD analysis was conducted. The XRD patterns for lower angles (2–10°) and higher angles (10–30°) are presented in Figure 5 and Figure 6, respectively.

As shown in Figure 5, Cloisite 10A presents a strong peak around 4.9°. This peak is related to the interlayer space [13,14,15,16,17], which is around 2 nm. The absence of such a peak in the composite fibers (both drawn and not drawn), indicates an exfoliated structure [18,19,20,21]. However, the content of the filler is low (1 and 2%), and a potential peak would not be easily detected. To further explore the existence of such a peak of Cloisite^®^ 10A in the composite fibers, an approach that is common for infrared spectrum manipulation was adopted. More specifically, the diffractogram of PP was subtracted from the diffractograms of the PP+1% Cloisite^®^ 10A samples (drawn and non-drawn fibers, see Figure S4 of the Supplementary Information File) and the Cl10A6 samples (drawn and non-drawn fibers, see Figure S5 of the Supplementary Information File). The subtracted diffractograms were multiplied by 10 in order to make a potential peak more visible. By this procedure, a peak around 4°, which is slightly broadened toward lower angles, can be just barely detected (see Figures S4 and S5 of the Supplementary Information File). The existence of the peak at lower angles (at 4° compared to 4.9° of the neat clay) reflects an intercalated structure, while the broadening of the peak reflects a partial exfoliation. Although this conclusion arises from a very weak XRD signal and should be viewed with skepticism, if it is accurate, it seems to contradict the mild negative effect of the clay on the tensile strength revealed in Figure 1, i.e., a rather acceptable dispersion resulting in the deterioration of mechanical properties. This point is further discussed with respect to the results for the other investigated clay in Section 4.3.

As shown in Figure 6, all samples containing PP presented peaks at 14, 17, 18.5, 21, and 22°. Those peaks are related to *a*-crystal planes (110), (040), (130), (111) and (131), and (041), respectively [22]. However, the weak peak at 16° and the medium peaks around 21° are not present in the drawn sample. Those two aforementioned peaks are related to the smectic phase of PP [23,24]. Higher cooling rates favor the formation of smectic forms in PP, and that was the case in this study, as the melted PP composite filaments exiting the extruder were immersed in an ice-water bath. According to the literature, the process of drawing induces the formation of *a*-crystals from the smectic phase [25]. Due to this effect, peaks around 20 to 25° were not present in samples stretched with a drawing ratio of 8 [25]. In another study, such peaks vanished in PP fibers that were annealed at 60 °C [23].

### 4.2. DoE with Cloisite^®^ 15A as a Filler

In Table 6, the factors and the responses for the DoE with Cloisite^®^ 15A as a filler are presented. The tensile strength and the onset decomposition temperature (T_dec_) were used as response variables.

#### 4.2.1. Main Effects of the Factors

**Figure 7:**
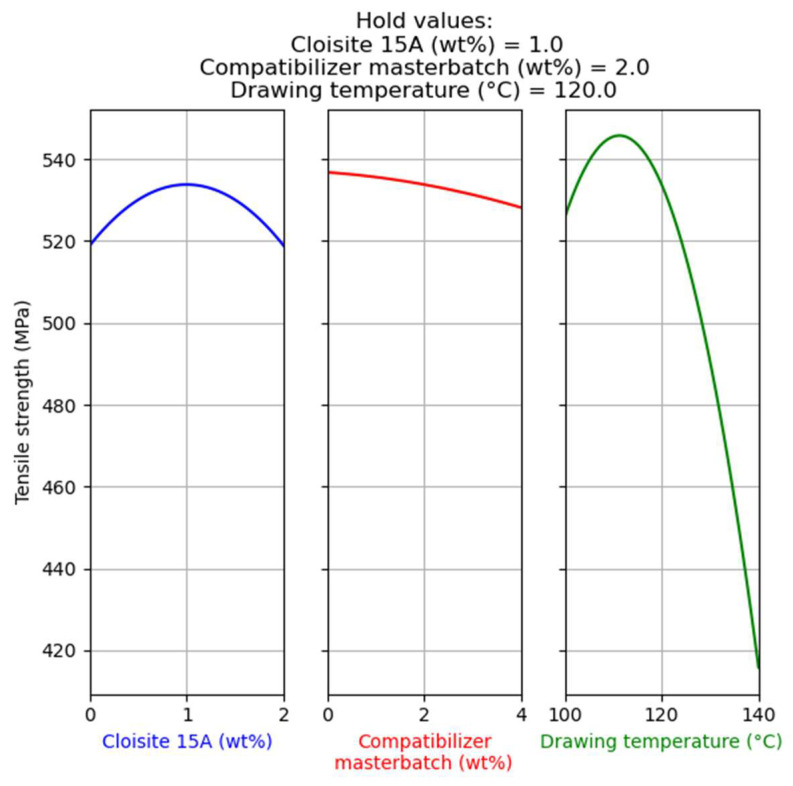
Main effect of factors on the tensile strength of composites with Cloisite^®^ 15A.

In Figure 7, the main factor effects on tensile strength are presented. (For more details about the way that such graphs are presented, see Section 4.1.1).

As presented in Figure 7, the filler and the compatibilizer content do not have a strong effect on tensile strength. On the other hand, the drawing temperature has a dominant effect on tensile strength and, similarly to the previous Design of Experiments results (Figure 1), there is a maximum of tensile strength at a drawing temperature of around 110 °C. However, a notable difference between these results and those shown in Figure 1 is that the addition of the filler does not have a negative impact on tensile strength (see the left plot of Figure 7).

The main factor effects on the onset decomposition temperature are presented in Figure S6 of the Supporting Information File. Similar to the previous DoE results (Figure S3 of the Supporting Information File), the results of Figure S6 reveal that none of the factors significantly contributes to the onset decomposition temperature (which deviates no more than approximately 10 °C) and, as will be shown later, the model that is created is not statistically significant. Consequently, this response variable will not be further investigated, and the rest of the study will focus on tensile strength.

#### 4.2.2. Combined Effect of the Factors

**Figure 8:**
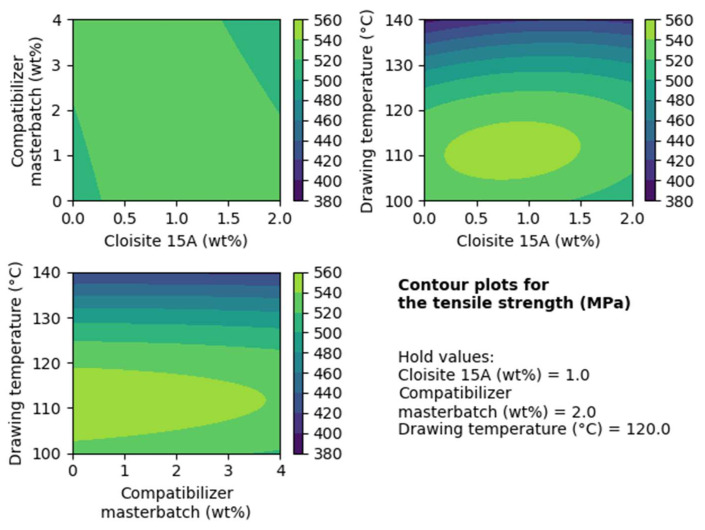
Contour plots for the tensile strength for the case of composites with Cloisite 15A.

In Figure 8, the contour plots for tensile strength are presented. In these plots, the effect on tensile strength of two out of three factors is presented while the third is kept constant (hold value).

Based on the upper graph on the right of Figure 8, it is apparent that filler contents between approximately 0.5–1 wt% and a drawing temperature around 110 °C give the highest tensile strength. Also, the lines formed from the different levels in all three contour plots of Figure 8 are almost parallel, indicating that there are not significant interactions among the factors that have a strong contribution to the tensile strength.

#### 4.2.3. Optimization

**Figure 9:**
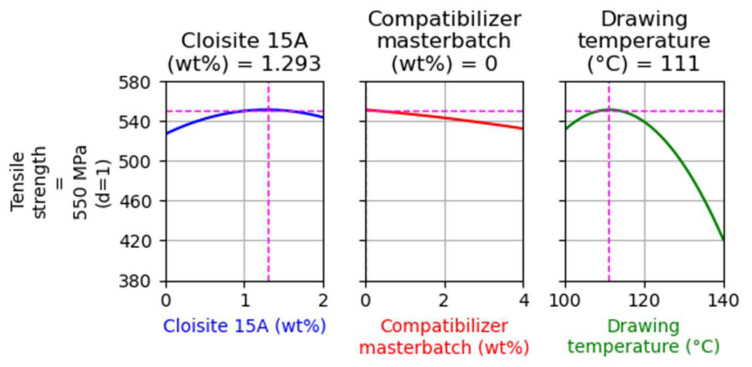
Optimization targeting the maximization of tensile strength without restrictions for the case of composites with Cloisite 15A.

**Figure 10:**
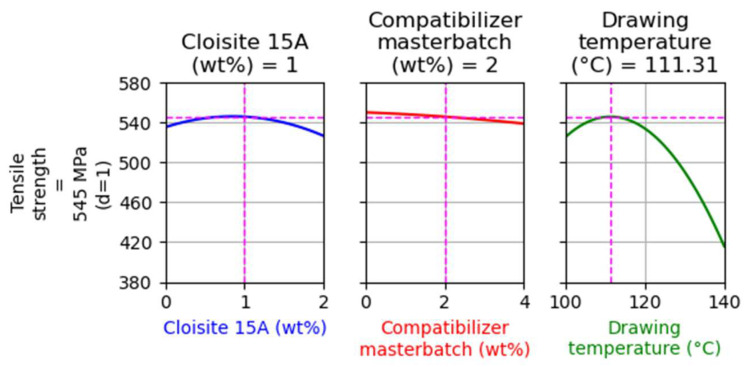
Optimization targeting the maximization of tensile strength, while the filler and the compatibilizer content were kept equal to 1 and 2% for the case of composites with Cloisite 15A.

Similarly to the previous DoE, two optimizations were performed, targeting the maximization of tensile strength. The first optimization had no restrictions, while the second was performed with the constrain that the filler and the compatibilizer content should be equal to 1 and 2 wt%, respectively. Such optimizations are presented in Figure 9 and Figure 10, respectively.

As expected, and as can be observed in Figure 9, zero compatibilizer content was estimated through the optimization, since the addition of the compatibilizer mainly has a negative effect on tensile strength. The model predicts that a fiber with 1.3 wt% filler, 0 wt% compatibilizer, and drawn at approximately 110 °C will present the maximum tensile strength, equal to 550 MPa.

In Figure 10, the results for the second optimization run are shown. The model predicts that fibers with 1 wt% filler, 2 wt% compatibilizer, and drawn at approximately 110 °C will present tensile strength equal to 545 MPa, which is a value very close to the maximum (550 MPa) shown by the optimization results of Figure 9. This sample was used to evaluate the model’s prediction ability.

#### 4.2.4. Model Evaluation

In order to confirm the prediction ability of the model, a sample with the composition shown in Figure 10 was produced and submitted to tensile tests and TGA. The same sample was also investigated using XRD before and after drawing. In Table 7, the experimental results and the model predictions are presented along with the relative deviations.

The relative absolute deviations between predictions and experimental results were low (around 12% for the tensile strength and 0% for the decomposition temperature). The statistical significance of the model is evaluated through the *p*-value and R^2^, which are presented in Table 8.

As it has been already mentioned, based on the *p*- and R^2^ values, the tensile strength model is statistically significant, while the model for the decomposition temperature presents poor statistical significance.

#### 4.2.5. Fiber Structure

**Figure 11:**
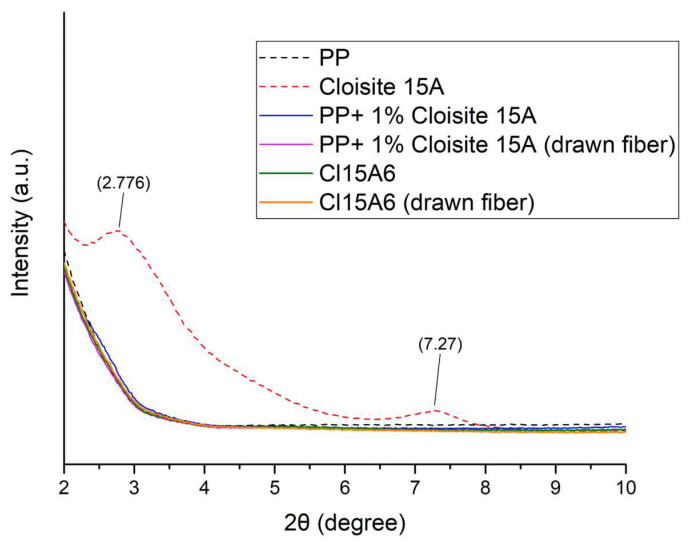
XRD diffraction patterns of PP, Cloisite^®^ 15A, two composite PP fiber samples containing 1 and 2% Cloisite^®^ 15A, drawn and not drawn, at lower angles (2–10°).

**Figure 12:**
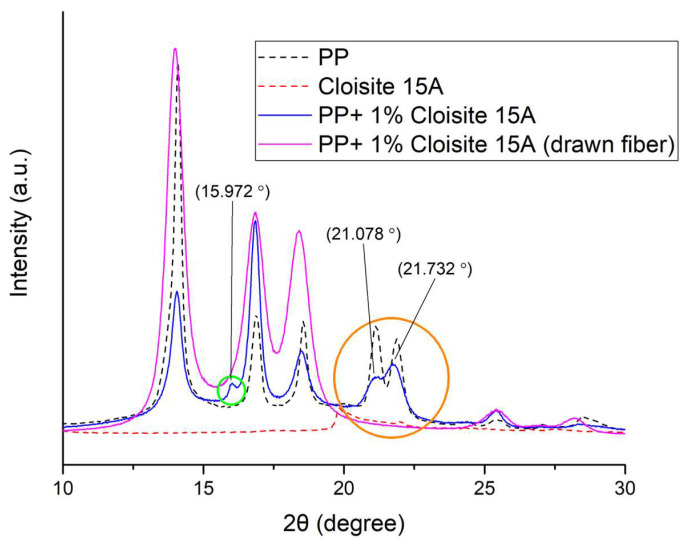
XRD diffraction patterns of PP, Cloisite^®^ 15A and a composite PP fibers containing 1% Cloisite^®^ 15A, drawn and not drawn, at higher angles (10–30°).

In order to investigate the structure of the drawn PP-Cloisite^®^ 15A fibers, XRD analysis was performed. The XRD figures for lower angles (2–10°) and higher angles (10–30°) are presented in Figure 11 and Figure 12, respectively.

As it is shown in Figure 11, Cloisite^®^ 15A presents two peaks around 2.8° and 7.3°. The first peak is related to the basal spacing (d_001_) of the organically modified clay, which is equal to 3.1 nm. The second, at 7.3°, corresponds to the interlayer distance of approximately 1.1 nm and it is related to the mineral that was not effectively modified or to the decomposition of the organic modifier during processing (the basal space of neat montmorillonite is around 1.2 nm). In the case of Cloisite^®^ 10A, the absence of such peaks in the XRD patterns of the composite fibers (both drawn and not drawn) shown in Figure 11 could indicate an exfoliated structure [18,19,20,21]. However, the content of the filler is low (1 and 2%), and a potential peak would not easily be detected. By performing the same diffractogram manipulation (subtraction of the PP curve from the respective curves of the PP+1 wt% Cloisite^®^ 15A for drawn and non-drawn fibers and multiplication by 10), which was carried out for the case of Cloisite^®^ 10A composites, interesting observations can be made (see Figures S7 and S8 of the Supplementary Information File). The peak at 7.3° does not exist in the subtracted curves, while the peak around 2.8° is shown at slightly lower 2θ values only for the non-drawn fibers (Figure S7). This observation suggests that only a minor intercalation was achieved, and the intercalation seems to have been reduced after drawing. Again, this conclusion should be approached with skepticism, but, if it is accurate, it reflects a poor dispersion of clay inside the polymer matrix. This observation is further discussed in Section 4.3 in relation to the tensile strength of the fibers.

As shown in Figure 12, at higher angles the results are similar to those presented and discussed for Figure 6 for the case of the Cloisite^®^ 10A composite PP fibers. The weak peak at 16° and the peaks around 21°, related to the smectic PP, are not present in the drawn fiber sample, since the drawing induces the formation of *a*-crystals from the smectic phase [25].

### 4.3. Further Discussion

Both designs revealed that the dominant factor that affects the tensile strength is the drawing temperature. In both cases, the optimum drawing temperature was found approximately 110 °C, which is rather lower than the temperature used in industrial applications (around 150 °C). However, the high speeds of fiber spinning and drawing in industry suggest that a steady state, and not a thermal equilibrium, is obtained, and, consequently, the actual drawing temperature is expected to be lower than the nominal value.

Trying to see the big picture, the clear conclusion of the analysis presented in the previous sections is that considering the increase of tensile strength, drawing is the dominating factor. The effects of clay and compatibilizer are extremely small compared to the effect of drawing. This is better understood by keeping in mind that the tensile strength of pristine (non-drawn) PP fibers is around 35 MPa [3,4], and that it goes to approximately 500 MPa (see for example the results for the first sample of Table 3) only when drawn with a ratio of 7 [3,4]. An increase of tensile strength by more than an order of magnitude is far higher than the improvement offered by any clay dispersed due to favorable intermolecular interactions inside a polymer matrix.

With this in mind, the question that needs to be answered is whether the addition of a clay further improves the tensile strength of drawn fibers.

The results of this study showed that the addition of Cloisite^®^ 15A up to approximately 1 wt% results in a small tensile strength enhancement, in contrast to Cloisite^®^ 10A, which shows a rather negative effect. Thus, as shown by the experimental results and the developed model based on those experiments, Cloisite^®^ 15A seems to perform better than Cloisite^®^ 10A.

The XRD signals of the composite PP fibers are rather weak at the characteristic of the clay 2θ values and, thus, no safe conclusion on the dispersion of both clays can be drawn. However, the manipulation of the XRD plots that was followed indicate a better dispersion of Cloisite^®^ 10A compared to that of Cloisite^®^ 15A. This is an observation with high uncertainty, but if it is true, it seems to contradict the tensile strength results, i.e., the clay with the better dispersion resulted in poorer tensile strength. This is a rather peculiar and unexpected conclusion, since in Composites Science the compatibility between matrix and additive is considered to be of major importance. Montmorillonite is modified for this reason. Compatibilizers are used for the same reason. More precisely, an increased wetting and contact surface between the matrix and the clay can contribute to a uniform stress distribution, which in turn leads to the absence of areas where accumulated stress would lead to the formation and growth of cracks. Also, chain entanglement around the particles and/or inside the interlayer space can contribute to the transfer of stresses in amorphous regions, which instead of breaking can dissipate large amounts of energy (stress) through plastic deformation and/or chain alignment. Such effects are well documented for various polymer-additive composites. Thus, results that show Cloisite^®^ 10A to be more compatible with PP than Cloisite^®^ 15A but cause worse mechanical properties are unexpected.

However, the above-mentioned contradiction can be understood if the above phenomena are considered in the context of drawing. A better dispersion of the clay prior to drawing increases the crystallinity of the matrix due to inhomogeneous nucleation [1]. In other words, the increased compatibility of Cloisite^®^ 10A with PP and the corresponding increased wetting and contact surface lead to more intense inhomogeneous nucleation, which in turn is responsible for high crystallization rates. However, it is well known that a smectic PP structure is desired prior to drawing, and that drawn fibers with better tensile strength are obtained from low crystallinity non-drawn fibers. For this reason, the fibers are rapidly cooled after they exit the extruder’s nozzle [1,3]. Drawing induces crystallization and, thus, a highly-crystallized structure prior to drawing interferes with the drawing process. Also, in some studies it is suggested that the introduction of the clay into the polymer matrix hinders the alignment of polymer chains during drawing [4]. In structures with good clay dispersion and increased wetting area (as in the case of Cloisite^®^ 10A), this hindering effect would be expected to be more intense. As mentioned above, drawing increases the tensile strength of PP from 35 MPa to around 500 MPa, an increase of more 1000%. Thus, it is expected that even a very minor effect in the drawing process would have a non-negligible impact on the properties of the fibers. In other words, any positive contribution from the particles can be easily negated if there is any interference with the drawing process. Thus, the effect of clays is not unequivocal, since they directly affect the properties of the composite similarly to the non-drawn samples, but also indirectly affect their properties by hindering drawing via various mechanisms (e.g., acting as nucleating agents and hindering chain alignment). Consequently, since drawing has a tremendous effect on the properties of the fibers, the indirect and non-obvious influences of particles prevail over their direct and expected influences on the properties of the drawn fibers.

The observation that an additive which results in the improvement of properties of non-drawn polymer samples shows an opposite effect in drawn fibers has been made also for other kinds of additives, such as a compatibilizer (PP-g-MA) and an antioxidant (combination of phosphite and phenolic types) [3,4]. For example, it was shown that an antioxidant is better dispersed in the polymer matrix when a compatibilizer is added, resulting in non-drawn samples with enhanced thermal and mechanical properties. However, upon drawing, the low molecular weight of the compatibilizer or the antioxidant results in a lower tensile strength compared to the neat PP fibers [3,4]. In addition, the antioxidant itself (independently of the interaction with the compatibilizer) has a dual effect: (a) due to its low molecular weight, it tends to deteriorate the drawing capability, and (b) due to the protection of the polymer from oxidative decomposition, it helps retain the initial molecular weight of PP, enhancing the effect of drawing (large chains present higher drawing potentials than short ones).

It is common in engineering and practical applications to consider the process a black box and to be interested only in the inputs and outputs. The concept of Design of Experiments has been developed to optimize complex processes in which multiple variables are involved and their influence on the output variables is not linear or independent of each other. For example, by increasing the compatibilizer content, a beneficial influence would be expected due to the enhancement of the antioxidant’s performance and the better dispersion of the particles. At the same time, a negative influence would be expected due to the antioxidant’s low molecular weight, which hinders the chain’s stretching/drawing potential. Thus, a maximum or a minimum in the tensile strength versus the compatibilizer content is expected to occur. However, the exact content at which the maximum (or minimum) tensile strength would be observed is not “universal” and may vary with the other input variables, such as the clay content, the drawing ratio, and temperature. In such a complex process, with competitive and synergistic effects among the various input variables, the analysis and discussion of experimental data can be performed on the basis of the models as presented in the previous sections.

Based on the above discussion, it should be pointed out that conclusions regarding polymer nanocomposites that are well documented for non-drawn polymer structures may not be valid for the anisotropic structures obtained from drawing.

## 5. Conclusions

In this study, two designs of experiments based on the Box-Behnken method were carried out, and the thermal and mechanical stability of polypropylene composite drawn fibers with two types of organically modified montmorillonite were optimized using a response surface methodology. In both designs, the filler content, specifically Cloisite^®^ 10A and Cloisite^®^ 15A for the first and the second design, respectively, the compatibilizer content and the drawing temperature were chosen as design variables/factors, while the tensile strength and the onset decomposition temperature were chosen as response variables.

However, the analysis for the onset decomposition temperature yielded a poor statistical model. Consequently, optimization runs were carried out only for the maximization of tensile strength. Samples were produced to evaluate the models’ prediction capabilities. It was concluded that both models perform satisfactorily.

Both designs revealed that the dominant factor that affects the tensile strength is the drawing temperature. The filler and the compatibilizer content did not show a pronounced effect. In both cases, the optimum drawing temperature was found to be approximately 110 °C, which is rather lower that the one used in industrial applications (approximately 150 °C), but the high speed of spinning and drawing in industry should be taken into account.

XRD analysis showed that drawing resulted in the transformation of the PP smectic phase to *a*-crystals. However, the clay characteristic XRD peaks in the diffractograms of the composite materials are weak, and no solid conclusion for the dispersion of both clays inside the polymer matrix can be derived. Nevertheless, the manipulation of the XRD diffractograms that followed suggests a better dispersion of Cloisite^®^ 10A compared to Cloisite^®^ 15A.

Furthermore, the results revealed that the addition of Cloisite^®^ 15A up to approximately 1 wt% enhances the tensile strength to a small extent, in contrast to Cloisite^®^ 10A, which shows a rather negative effect. In other words, the clay with the better dispersion resulted in poorer tensile strength of drawn fibers, which can be explained by the higher crystallinity that the better clay dispersion induces (which is undesirable prior to drawing) and the potential hindering of polymer chain alignment during drawing induced by large clay platelets.

Overall, this study shows that conclusions regarding polymer nanocomposites that are well documented for non-drawn polymer structures may not be valid for the anisotropic structures obtained from drawing.

## Figures and Tables

**Table 1:** Important characteristics of the used materials.

Material	Trade Name	Characteristics ^1,2,3^	Supplier
Isotactic PP	Ecolen HZ42Q	MFI = 18 g/10 min, TS = 33 MPa, T_m_ = 168–171 °C	Hellenic Petroleum S.A., Thessaloniki, Greece
Masterbatch with compatibilizer	Bondyram^®^ 1001	PP grafted with maleic anhydride (PP-g-MA). MA content 1%, MFI = 100 g/10 min, T_m_ = 160 °C	Polyram Plastic Industries Ltd., Gilboa, Israel
Masterbatch with antioxidant	KRITILEN^®^ AO PP9216	PP with 20.5 wt.% antioxidant (combination of phosphite and phenolic types)	Plastika Kritis S.A., Heraklion, Greece
Organically modified montmorillonite	Cloisite^®^ 10A	Montmorillonite treated with quaternary ammonium salts (2Μ2HΤ), CEC = 125 meq/100 g	Southern Clay Products Inc. (Austin, TX, USA)
Organically modified montmorillonite	Cloisite^®^ 15A	Montmorillonite treated with quaternary ammonium salts (2ΜBHΤ), CEC = 125 meq/100 g	Southern Clay Products Inc. (Austin, TX, USA)

^1^ MFI: melt flow index, ^2^ TS: tensile strength, ^3^ T_m_: melting point.

**Table 2:** Experimental matrix for both designs.

Sample Name	Cloisite^®^ (wt%)	Compatibilizer Masterbatch (wt%)	Drawing Temperature (°C)
Cl1	0	0	120
Cl2	2	0	120
Cl3	0	4	120
Cl4	2	4	120
Cl5	0	2	100
Cl6	2	2	100
Cl7	0	2	140
Cl8	2	2	140
Cl9	1	0	100
Cl10	1	4	100
Cl11	1	0	140
Cl12	1	4	140
Cl13	1	2	120
Cl14	1	2	120
Cl15	1	2	120

**Table 3:** Factors and response variables for the DoE with Cloisite^®^ 10A.

Sample	Cloisite^®^ (wt%)	Compatibilizer Masterbatch (wt%)	Drawing Temperature (°C)	Tensile Strength (MPa)	*T_dec_* (°C)
Cl10A1	0	0	120	506 ± 34	294
Cl10A2	2	0	120	472 ± 39	296
Cl10A3	0	4	120	515 ± 22	290
Cl10A4	2	4	120	468 ± 40	296
Cl10A5	0	2	100	536 ± 34	302
Cl10A6	2	2	100	496 ± 37	296
Cl10A7	0	2	140	384 ± 29	281
Cl10A8	2	2	140	387 ± 25	285
Cl10A9	1	0	100	490 ± 29	293
Cl10A10	1	4	100	468 ± 33	298
Cl10A11	1	0	140	449 ± 24	298
Cl10A12	1	4	140	365 ± 23	301
Cl10A13	1	2	120	511 ± 23	301
Cl10A14	1	2	120	500 ± 27	290
Cl10A15	1	2	120	510 ± 25	297

**Table 4:** Tensile test and TGA experimental results compared to theoretical predictions.

	Tensile Strength (MPa)	Decomposition Temperature (°C)
Validation sample	481 ± 28	291
Optimization prediction	519	297
Absolute relative deviation (%)	7.4	2

**Table 5:** R^2^ and *p*-value for the tensile strength and the decomposition temperature statistical models.

	Tensile Strength Model	Decomposition Temperature Model
R^2^ (%)	90.63	40.76
*p*-value	0.039	0.900

**Table 6:** Factors and response variables for the DoE with Cloisite^®^ 15A.

Sample	Cloisite^®^ (wt%)	Compatibilizer Masterbatch (wt%)	Drawing Temperature (°C)	Tensile Strength (MPa)	T_dec_ (°C)
Cl15A1	0	0	120	506 ± 34	294
Cl15A2	2	0	120	543 ± 25	289
Cl15A3	0	4	120	515 ± 22	290
Cl15A4	2	4	120	500 ± 31	282
Cl15A5	0	2	100	536 ± 34	302
Cl15A6	2	2	100	505 ± 31	293
Cl15A7	0	2	140	384 ± 29	281
Cl15A8	2	2	140	393 ± 24	291
Cl15A9	1	0	100	546 ± 28	294
Cl15A10	1	4	100	509 ± 21	297
Cl15A11	1	0	140	421 ± 35	296
Cl15A12	1	4	140	365 ± 23	300
Cl15A13	1	2	120	540 ± 23	295
Cl15A14	1	2	120	518 ± 33	289
Cl15A15	1	2	120	539 ± 39	300

**Table 7:** Tensile test and TGA experimental results compared with theoretical predictions.

	Tensile Strength (MPa)	Decomposition Temperature (°C)
Validation sample	478 ± 39	296
Optimization prediction	545	296
Relative absolute deviation (%)	12.4	0

**Table 8:** R^2^ and *p*-value for the Tensile strength and decomposition temperature statistical models.

	Tensile Strength (MPa)	Decomposition Temperature (°C)
R^2^ (%)	96.19	56.97
*p*-value	0.005	0.676

## Data Availability

Data are contained within the article.

## Supplementary Materials

The following supporting information can be downloaded at: https://www.mdpi.com/article/10.3390/nano14020223/s1, Figure S1: Typical stress-strain curves (see Table 3 and Table 6 of the main file for the composition of the Cl10A13 and Cl15A4 samples, respectively); Figure S2: Typical TGA curves (see Table 3 and Table 6 of the main file for the composition of the Cl10A3 and Cl15A8 samples, respectively); Figure S3: Main effect of factors on decomposition temperature regarding the composites with Cloisite^®^ 10A; Figure S4: XRD diffractograms of Cloisite^®^ 10A, and subtracted diffractogram of PP+1% Cloisite^®^ 10A (drawn and non-drawn fibers) minus PP. The subtracted diffractogram was multiplied by 10; Figure S5: XRD diffractograms of Cloisite^®^ 10A, and subtracted diffractogram of the Cl10A6 samples (drawn and non-drawn fibers) minus PP. The subtracted diffractogram was multiplied by 10; Figure S6: Main effect of factors on decomposition temperature, regarding the composites with Cloisite^®^ 15A; Figure S7: XRD diffractograms of Cloisite^®^ 15A, and subtracted diffractogram of PP+1% Cloisite^®^ 15A (drawn and non-drawn fibers) minus PP. The subtracted diffractogram was multiplied by 10; Figure S8: XRD diffractograms of Cloisite^®^ 15A, and subtracted diffractogram of the Cl15A6 samples (drawn and non-drawn fibers) minus PP. The subtracted diffractogram was multiplied by 10.
