# Supplementary material for: Properties Optimization of Polypropylene/Montmorillonite Nanocomposite Drawn Fibers
Source: Nanomaterials (Basel). 2024 Jan 19;14(2):223. doi: 10.3390/nano14020223 (PMC10819339; doi:10.3390/nano14020223)
Supplement: Supplementary file 1 [file nanomaterials-14-00223-s001.zip › nanomaterials-2791194-supplementary.pdf]

nanomaterials

*Supplementary Information for*

# Properties Optimization of Polypropylene/Montmorillonite Nanocomposite Drawn Fibers

**Konstantinos Leontiadis [1], Katerina Theodoratou [1], Costas Tsioptsias [2] and Ioannis Tsivintzelis [1,*]**

[1] Department of Chemical Engineering, Aristotle University of Thessaloniki, University Campus, 54124 Thessaloniki, Greece
[2] Department of Chemical Engineering, University of Western Macedonia, 50132 Kozani, Greece
* Correspondence: tioannis@cheng.auth.gr

**Citation:** Leontiadis, K.; Theodoratou, K.; Tsioptsias, C.; Tsivintzelis, I. Properties Optimization of Polypropylene/Montmorillonite Nanocomposite Drawn Fibers. *Nanomaterials* **2024**, *14*, 223. https://doi.org/10.3390/nano14020223

Academic Editor: Boris Mahltig

Received: 9 December 2023
Revised: 12 January 2024
Accepted: 16 January 2024
Published: 19 January 2024

**Copyright:** © 2024 by the authors. Licensee MDPI, Basel, Switzerland. This article is an open access article distributed under the terms and conditions of the Creative Commons Attribution (CC BY) license (https://creativecommons.org/licenses/by/4.0/).

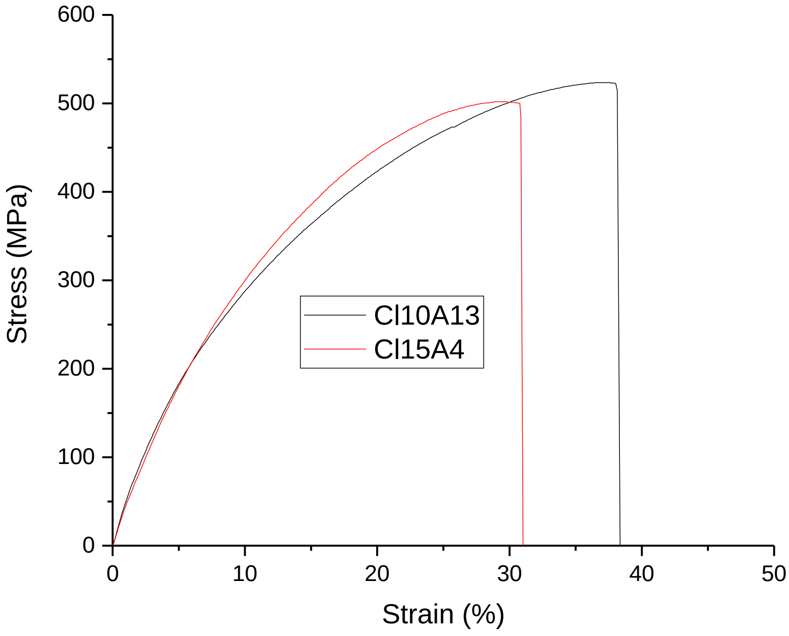


**Figure S1.** Typical stress-strain curves (see Tables 3 and 6 of the main file for the composition of the Cl10A13 and Cl15A4 samples, respectively).

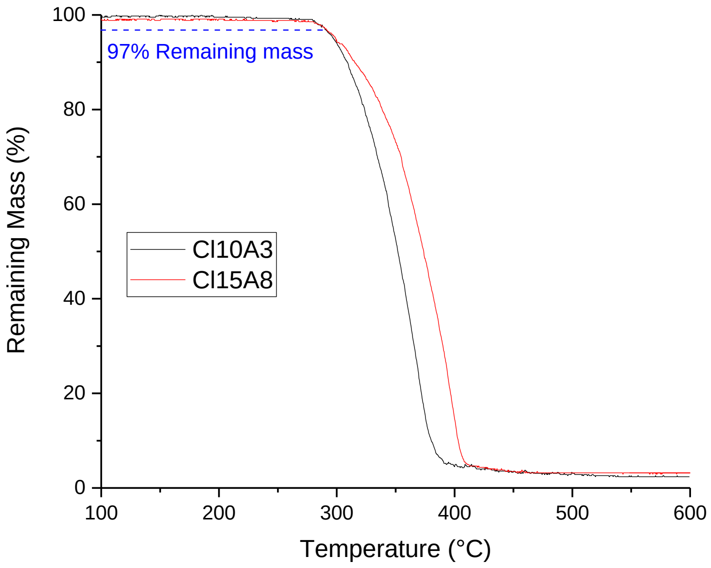


**Figure S2.** Typical TGA curves (see Tables 3 and 6 of the main file for the composition of the Cl10A3 and Cl15A8 samples, respectively).

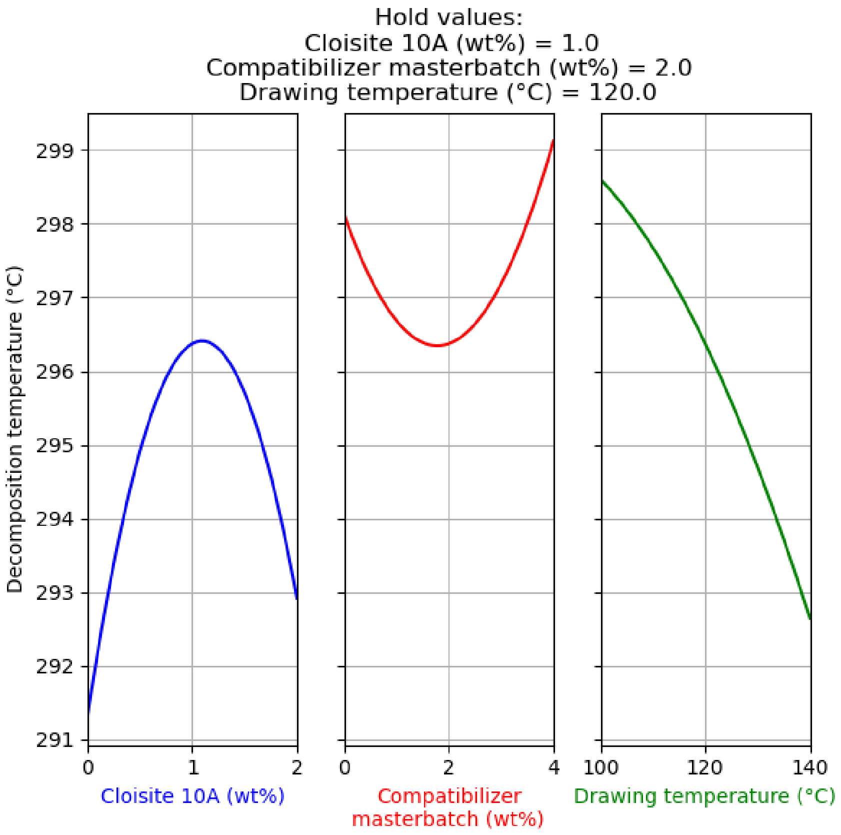


**Figure S3.** Main effect of factors on decomposition temperature regarding the composites with Cloisite® 10A.

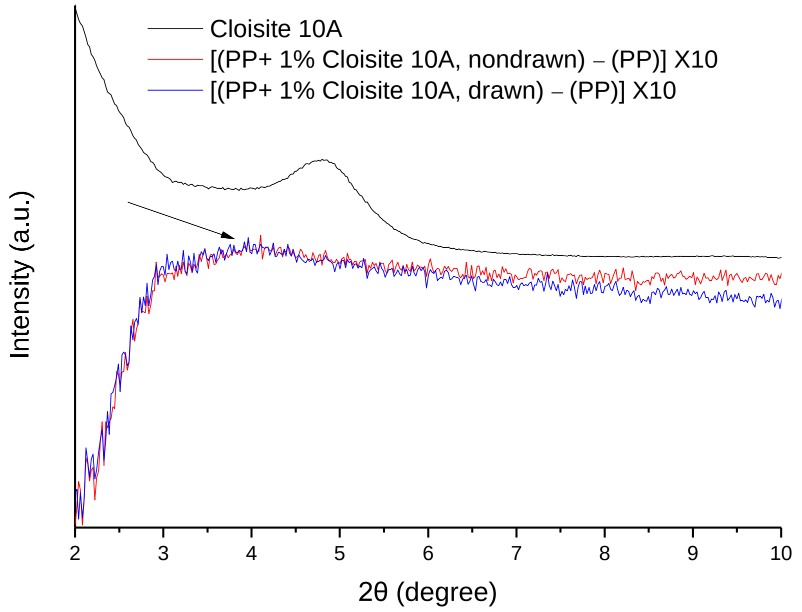


**Figure S4.** XRD diffractograms of Cloisite 10A, and subtracted diffractogram of PP+1% Cloisite® 10A (drawn and non-drawn fibers) minus PP. The subtracted diffractogram was multiplied by 10.

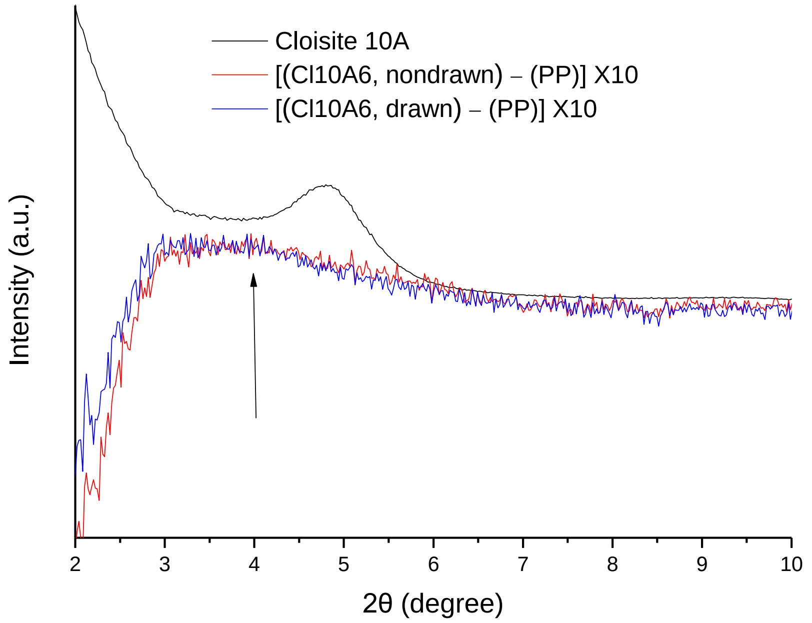


**Figure S5.** XRD diffractograms of Cloisite® 10A, and subtracted diffractogram of the Cl10A6 samples (drawn and non-drawn fibers) minus PP. The subtracted diffractogram was multiplied by 10.

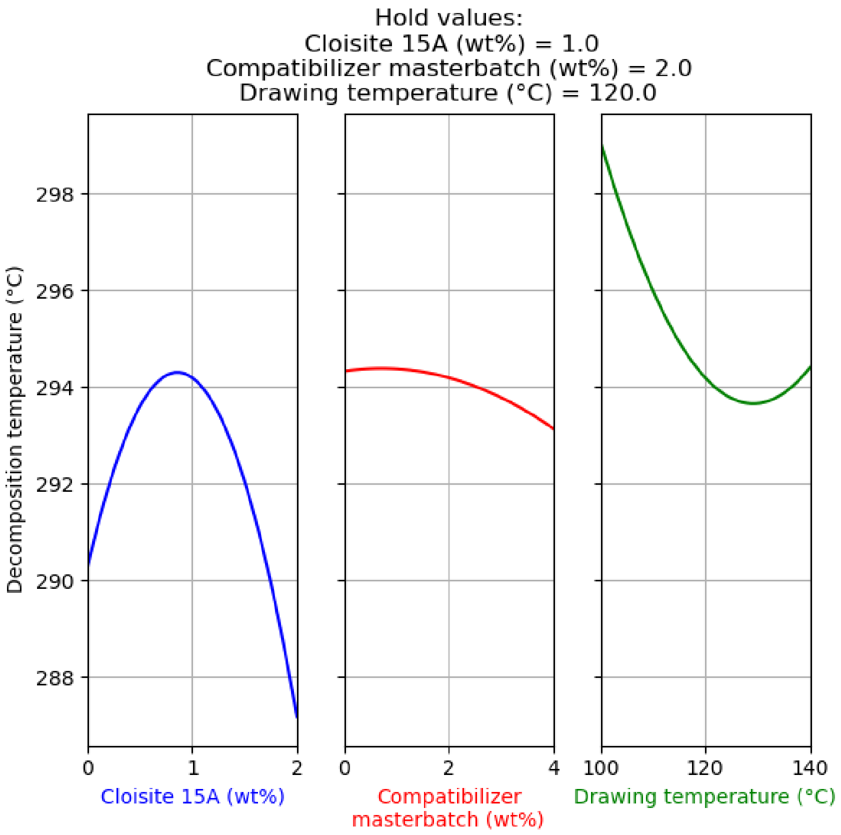


**Figure S6.** Main effect of factors on decomposition temperature, regarding the composites with Cloisite® 15A.

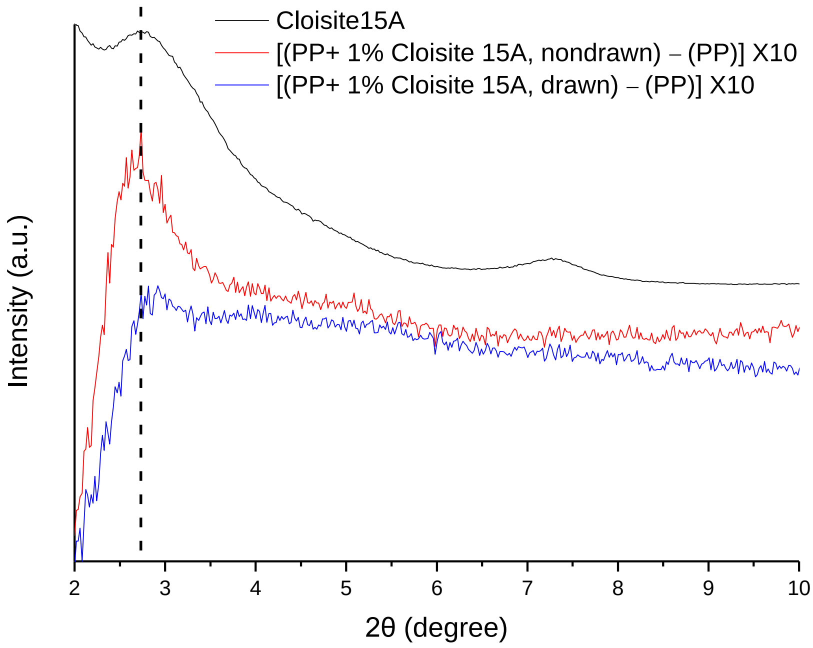


**Figure S7.** XRD diffractograms of Cloisite® 15A, and subtracted diffractogram of PP+1% Cloisite® 15A (drawn and non-drawn fibers) minus PP. The subtracted diffractogram was multiplied by 10.

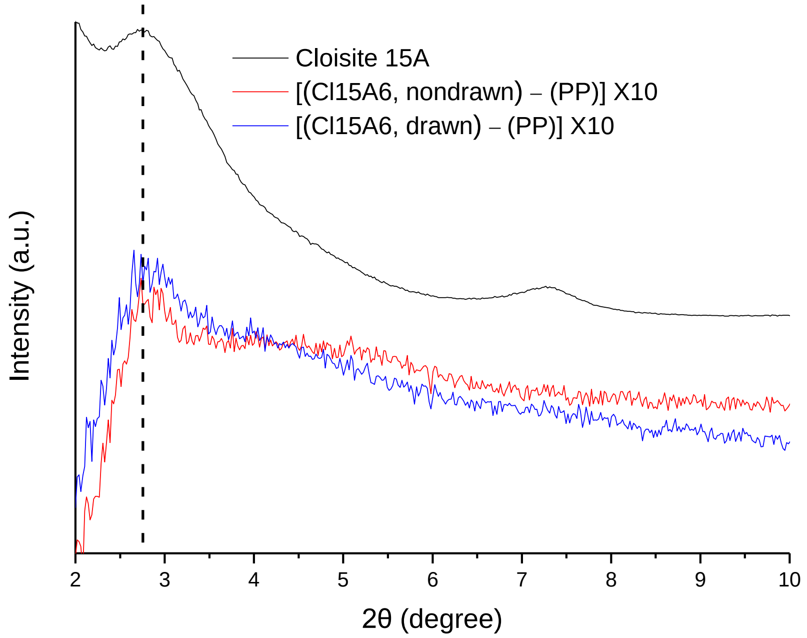


**Figure S8.** XRD diffractograms of Cloisite® 15A, and subtracted diffractogram of the Cl15A6 samples (drawn and non-drawn fibers) minus PP. The subtracted diffractogram was multiplied by 10.
